# Prevention of Infections in Early Life: Advances, Challenges and Future Directions

**DOI:** 10.3390/vaccines14070585

**Published:** 2026-06-30

**Authors:** Eleni Vergadi

**Affiliations:** Department of Paediatrics, Medical School, University of Crete, 71500 Heraklion, Greece; eleni.vergadi@uoc.gr

## 1. Introduction

The early stages of life represent a critical window that shapes health and well-being throughout the lifespan [1]. During pregnancy, delivery, and the early postnatal period, foetuses, neonates, and young infants are particularly susceptible to a wide range of infections as their yet-untrained and undeveloped immune system is limited in capacity to mount robust immune responses [1,2].

Over the past decade, the field of early-life infection prevention has profoundly transformed. Maternal immunisation has emerged as one of the most effective strategies for protecting neonates and young infants during the first months of life, particularly from infections such as respiratory syncytial virus (RSV), influenza, pertussis, tetanus, and COVID-19, when direct vaccination is unavailable or infant vaccination has not yet provided optimal protection. [3]. Simultaneously, novel preventive strategies have expanded to include long-acting monoclonal antibodies against RSV and the ongoing development of maternal vaccines targeting Group B *Streptococcus* (GBS) and Cytomegalovirus (CMV) [3,4]. In addition, the introduction of higher-valency pneumococcal conjugate vaccines as well as advances on vaccine design, delivery systems, and public health implementation strategies continue to reshape the field [3,4,5].

Despite these advances, young infants continue to experience a disproportionate burden of infectious diseases; infectious diseases account for nearly half the deaths of all those under 5 years of age globally [6]. The implementation challenges remain substantial; vaccine hesitancy, inequitable access to immunisation services, disruptions to vaccination programmes during public health emergencies, and persistent disparities among countries continue to limit the impact of otherwise highly effective preventive interventions [7,8,9]. Furthermore, vulnerable populations, including premature infants, immunocompromised children, and transplant recipients, often require tailored approaches that are not adequately addressed by conventional vaccination schedules.

This Special Issue, “Vaccines and Prevention of Infections in Early Life”, addresses several of these challenges through contributions spanning maternal vaccination, vaccine implementation, health policy, vaccine access, and optimisation of immunisation practices.

## 2. Maternal and Infant Protection

One of the major themes emerging from this Special issue is the importance of maternal strategies for protecting infants during the first months of life. However, vaccine uptake during pregnancy remains suboptimal in many settings. Lis-Kuberka and Orczyk-Pawiłowicz highlight the ongoing problem of suboptimal maternal vaccination uptake, with only a minority of Polish women receiving influenza and COVID-19 vaccines during pregnancy [10]. The authors provide insights into the factors associated with vaccine acceptance and emphasise the critical role of healthcare professionals in promoting maternal immunisation and breastfeeding.

The importance of maternal immunisation is further underscored by the review of pertussis resurgence by Nian et al. [11]. Despite decades of successful vaccination programmes, pertussis continues to re-emerge globally, particularly threatening newborns and young infants who are too young to be fully protected through routine vaccination schedules. The authors discuss several factors contributing to this resurgence, including waning immunity, pathogen adaptation, incomplete vaccine coverage, and the limitations of the current acellular pertussis vaccines. Nian et al. emphasise the importance of optimised vaccination strategies, including maternal immunisation during pregnancy, booster vaccination programmes, and the development of next-generation pertussis vaccines capable of inducing more durable and effective protection [11].

## 3. Vaccine Implementation, Access, and Equity

Scientific advances alone are insufficient to reduce the burden of infectious diseases if vaccines remain inaccessible or underutilised. Several contributions in this Special Issue address the broader public health and policy factors that influence vaccine uptake. Ma et al. examined access to DTP-based combination vaccines across ten Asia–Pacific countries between 2019 and 2022 [12]. Their analysis revealed disparities in vaccine availability, affordability, and accessibility, with lower-income countries experiencing more disruption during the COVID-19 pandemic. The study highlights the vulnerability of immunisation systems during public health emergencies and emphasises the need to strengthen vaccine supply chains and routine immunisation infrastructure to ensure equitable vaccine access [12].

Economic barriers continue to represent major obstacles to vaccine uptake worldwide. Malchrzak et al. provide an important example of how vaccine policy influences vaccination behaviour [13]. Following the introduction of a universal free pneumococcal vaccination in Poland, the uptake of other recommended vaccines also significantly increased. Removing financial barriers increased the uptake not only of pneumococcal vaccination but also of other recommended vaccines. These observations suggest that reducing financial barriers has broader positive effects on vaccine confidence and acceptance. Together, these studies highlight that the success of vaccination programmes depends on not only on vaccine availability but also equitable access, sustainable financing, and effective implementation.

## 4. Optimising Vaccination Practices

As immunisation programmes continue to evolve, attention is also being directed toward optimising vaccine delivery and tailoring vaccination strategies to specific populations. One frequently overlooked aspect of vaccination is the vaccination experience. The pain associated with infant immunisation can contribute to parental anxiety and negatively influence future attitudes towards vaccination. In their systematic review, Pop et al. demonstrate that topical lidocaine–prilocaine significantly reduces pain scores and behavioural indicators of distress during infant immunisation [14]. Improving the vaccination experience represents an important strategy for enhancing parental satisfaction and supporting vaccine adherence.

In an era of declining herd immunity and the resurgence of vaccine-preventable diseases in several settings worldwide, another important challenge involves protecting immunocompromised children. Hartley et al. review the evidence regarding the safety and efficacy of measles, mumps, rubella (MMR), and varicella vaccines in selected paediatric solid-organ transplant recipients and discuss practical considerations for vaccine administration before and after transplantation [15]. Their work reflects a broader trend towards more individualised vaccination strategies that balance risks and benefits according to patient-specific characteristics.

## 5. Conclusions and Future Directions

The landscape of early-life infection prevention is rapidly evolving. Recent advances in RSV preventive strategies and the ongoing development of maternal vaccines against GBS and CMV have the potential to further reduce the burden of infectious diseases in neonates and young infants. Additionally, precision vaccinology, systems immunology and immunogenetics are improving our understanding of vaccine responses during early life and may enable more individualised preventive strategies. Although remarkable progress has been achieved, challenges remain, including suboptimal vaccine uptake, persistent disparities in access to vaccines, and the need for tailored approaches in high-risk populations.

Ultimately, the future of early-life infection prevention will depend not only on the development of new vaccines and technologies but also on the successful translation of existing knowledge into equitable and effective public health practice. Future research should focus on developing novel vaccines and immunisation approaches as well as identifying effective strategies to increase vaccine confidence in pregnancy, uptake, and equitable access across diverse populations.
